# Influence of Irrigant Activation Techniques on External Root Temperature Rise and Irrigation Penetration Depth in 3D-Printed Tooth Model: An In Vitro Study

**DOI:** 10.3390/dj13070295

**Published:** 2025-06-29

**Authors:** Ali Addokhi, Ahmed Rahoma, Neveen M. A. Hanna, Faisal Alonaizan, Faraz Farooqi, Shimaa Rifaat

**Affiliations:** 1 Endodontic Fellowship Program, College of Dentistry, Imam Abdulrahman Bin Faisal University, Dammam 31441, Saudi Arabia; 2 Department of Restorative Dental Sciences, College of Dentistry, Imam Abdulrahman Bin Faisal University, Dammam 31441, Saudi Arabiasrhussein@iau.edu.sa (S.R.); 3 Department of Dental Education, College of Dentistry, Imam Abdulrahman Bin Faisal University, Dammam 31441, Saudi Arabia; fafarooqi@iau.edu.sa

**Keywords:** irrigation, temperature, activation, biofilm, 3D-printed teeth, ultrasonic, sonic, diode laser

## Abstract

**Introduction:** Successful root canal therapy relies on thorough cleaning and disinfection to eliminate microorganisms and residual pulp tissue. Advanced irrigation activation techniques, including Sonic, Ultrasonic, and Diode Laser activation, have improved cleaning efficacy, bacterial reduction, smear layer removal, and irrigant hydrodynamics. On the other hand, these irrigation activation techniques may lead to a temperature rise that may risk the surrounding periodontal tissue. Thus, this study aimed to investigate the temperature rise during different irrigation activation techniques at various time intervals and evaluate the efficacy of these techniques in removing biofilm-mimicking hydrogel BMH of a simulated root canal system in 3D-printed tooth models. **Methods:** Ten extracted human mandibular premolars, prepared to size 40/0.04 taper, and a hundred 3D-printed resin premolars with simulated main (0.25 mm) and lateral canals (0.15 mm at 3, 7, 11 mm from apex) were used; 50 of them were filled with biofilm-mimicking hydrogel (BMH). Five irrigation activation techniques were evaluated: Diode Laser, Ultrasonic, Sonic, XP-Finisher, and Control (*n* = 10). Temperature rises were measured on the extracted premolars after 30 and 60 s of activation using a thermographic camera in a controlled environment (23 ± 2 °C). Irrigant penetration, with and without BMH, was assessed in 3D-printed premolars using a 2.5% sodium hypochlorite-contrast medium mixture, visualized with a CMOS radiographic sensor. Penetration was scored (main canal: 3 points; lateral canals: 0–2 points) and analyzed with non-parametric tests. **Results:** Diode Laser activation technique resulted in the highest temperature rise on the external root surface, followed by the Ultrasonic, with no statistically significant difference observed among the remaining groups. In terms of efficacy, Ultrasonic and Sonic activation achieved significantly greater irrigant penetration in samples without BMH, and greater BMH removal in samples with BMH, compared to Diode Laser, XP-Finisher, and Control groups. **Conclusions:** In this in vitro study, Diode Laser caused the highest temperature rise, followed by Ultrasonic, with significant increases from 30 to 60 s. Temperature rise did not significantly affect penetration or BMH removal. Ultrasonic and Sonic irrigation techniques achieved the highest depth of penetration (without BMH) and biofilm-mimicking Hydrogel removal (with BMH) compared to Diode Laser, XP-Finisher, and Control.

## 1. Introduction

The success of root canal therapy relies on thorough cleaning and disinfection to eliminate microorganisms, residual pulp tissue, and bacterial nutrients. During endodontic treatment, root canal enlargement aims to mechanically remove infected dentin and enhance irrigant penetration for improved disinfection [1,2]. Despite advancements in endodontic files, studies estimate that 10–80% of dentinal walls remain untouched, depending on file design and canal anatomy [3]. The prevalence of lateral canals ranges from 27.4% to 99%. These anatomies may harbor tissue and bacteria that can potentially reside in lateral canals [4,5,6,7]. Advanced irrigation activation techniques, including Sonic, Ultrasonic, and Diode Laser techniques, have been developed to enhance cleaning efficacy and irrigant penetration into these anatomical variations [8,9,10].

Many studies investigated irrigation activation techniques (Sonic, Ultrasonic, and Diode Laser) in terms of heat generation, which can elevate root surface temperatures [11,12,13,14], potentially risking damage to surrounding bone and periodontal tissues, with damage reported at temperatures above 47 °C [15,16,17]. One study reported that the ultrasonic endodontic device caused necrosis of the alveolar bone, and thermal damage was observed in the periodontal ligament in the upper anterior region. Additionally, there was an inflammatory reaction in the adjacent nasal cavity, which resulted in the loss of all upper incisors [18].

Diode Laser has been introduced in endodontics as an adjunctive irrigation activation technique. Diode Laser irradiation has been shown to effectively remove the smear layer and debris from root canal walls, enhancing irrigant penetration and overall cleaning efficacy [19]. The 810 nm Diode Laser provides lower temperature rise but superior bacterial reduction, while the 980 nm diode laser exhibits significant temperature rise, making it efficient for the ablation and vaporization of soft tissue, due to its enhanced water absorption properties [20,21].

One of the new modalities that FKG Dentaire has introduced as an irrigant activation device is the XP-Endo Finisher, which is an innovative nickel–titanium (NiTi) file designed for enhanced root canal cleaning [22,23,24]. 

The utilization of three-dimensional (3D)-printed teeth in endodontic research offers significant advantages over natural teeth in terms of replicating the anatomical features of natural teeth using cone beam computed tomography (CBCT) or micro-CT data, which aids in providing a standardized model that ensures consistent morphology across samples, thereby enhancing the reliability of experimental results [25,26].

To the author’s knowledge, there are no studies on the temperature rise on dual-wavelength diode laser incorporating dual wavelengths of (810 + 980 nm) and the XP-Finisher operating at 1000 rpm. Furthermore, few studies have investigated irrigant penetration into a standardized simulated root canal system, reporting qualitative results that inherently had limited reproducibility [27,28]. Quantifying the depth of penetration into the simulated lateral canal provides precise, reproducible data over qualitative assessments [29].

Thus, this study aims to evaluate temperature rise on the outer root surface of extracted premolar teeth and irrigant penetration into artificially created main and lateral canals in 3D-printed resin tooth models using five activation techniques: Sonic, Ultrasonic, Dual-Wavelength Diode Laser, XP-Finisher, and a control. The null hypotheses are (1) H_01_: There are no significant differences in temperature rise on the outer root surface among irrigation activation techniques (Control, Diode Laser, Ultrasonic, Sonic, XP-Finisher) at 30 s and 60 s, and (2) H_02_: There are no significant differences in total scores for depth of penetration of irrigation mixture and Biofilm-Mimicking Hydrogel removal among irrigation activation techniques (Control, Diode Laser, Ultrasonic, Sonic, XP-Finisher).

## 2. Materials and Methods

This study was approved by the Institutional Review Board (IRB-PGS-2024-02-419) of Imam Abdulrahman Bin Faisal University (IAU), ensuring compliance with ethical standards for research involving human-derived samples.

**Sample size Determination:**

The sample size was determined using an online calculator (http://statulator.com/SampleSize/ss2M.html, accessed on 17 May 2025) with an alpha value of 0.05 and 95% power. The means (33 and 27.33 units) and pooled standard deviation (2.77 units) were obtained from a relevant previous study evaluating temperature rise during irrigation activation on the outer root surface [29]. The calculator determined that a minimum sample size of 8 teeth per group was required for a two-sample comparison (e.g., Diode Laser vs. Control), which was increased to 10 to account for potential sample loss (*n* = 10).

**(I) Temperature rise analysis:**

 **(i)**  **Sample selection:**

Ten extracted single-rooted human mandibular premolar teeth, extracted for orthodontic reasons from patients aged 17–25 years. Teeth were stripped of soft tissue and sterilized by immersion in 2.5% NaOCl for 72 h [30] and scaled using an ultrasonic scaler if calculus was visible. After washing with water for 2 min, the inclusion criteria include single, straight root canals with minimal curvature (0–12°), complete root formation without any internal or external resorption. Samples selected with a length of 22 mm ± 1. In a way to standardize the root dentin thickness, the samples were subjected to cone-beam computed tomography (CBCT) using a CS 9300 machine (Carestream Dental, New York, NY, USA) at 70 kV, 5.0 mA, and 90 μm voxel size to ensure homogeneity of the samples and reduce differences in dentinal thickness effect on the temperature rise [31]. Dentinal thickness was measured in axial view at the cervical, middle, and 3 mm from the root apex in bucco-lingual and mesio-distal directions (Table 1). The selected teeth were stored in 0.1% thymol at 5 degrees until the time of the experiment.

 **(ii)**  **Sample preparation:**

The ten samples were used to evaluate temperature rise during irrigation activation for 30 s followed by 60 s activation durations.

**Figure 1:**
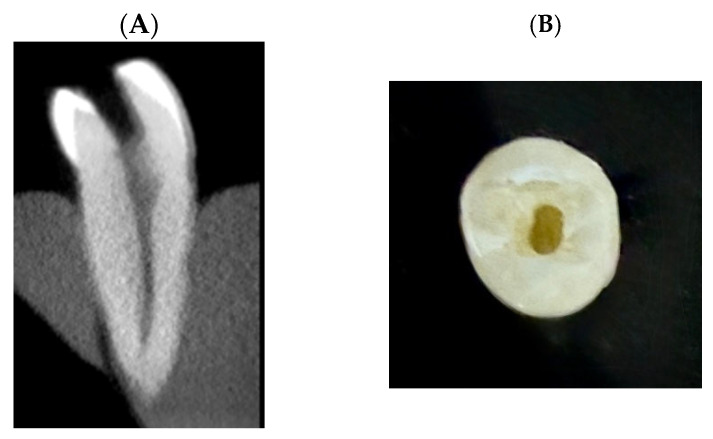
Representative sample showing the standardized access cavity preparation. All samples received a traditional endodontic access cavity to allow consistent canal instrumentation and irrigation. (**A**): CBCT Sagittal view of selected sample. (**B**): Occlusal photograph of the same sample showing the traditional access cavity.

A traditional access cavity has been created in all samples using a diamond bur—845KRD.314.025 (Komet Dental, Lemgo, Germany) (Figure 1) [32]. Patency was established using an ISO 10 C-Pilot file (VDW GmbH, Munich, Germany) under an operating microscope (A6 series, Global Surgical Corporation, St. Louis, MO, USA). Working length has been determined when the tip of the file was visible at the apex, 1 mm subtracted to estimate the working length of each sample. Root canals were prepared with vortex rotary files (Dentsply Sirona, Tulsa, OK, USA) to size 40/0.04 taper per manufacturer’s instructions. A total of 2 mL of irrigant with 2.5% sodium hypochlorite (Endo-Access, France) using a 27-gauge side-vented needle (GoldenDent, Roseville, MI, USA) after each file. The canal was then dried using a paper point (PD, dentire SA, La Chaux-de-Fonds, Switzerland).

 **(iii)**  **Grouping:**

A sample of 10 teeth was used across five groups to evaluate temperature rise during irrigation activation [29], with the same 10 teeth tested for all groups to minimize sample variability. The groups employed distinct activation techniques:

Group A—Diode Laser: Dual-Wavelength Diode Laser 810 + 980 nm, 0.7 W, 12 W Dual 4″ (Quicklase, Canterbury, UK).

Group B—Ultrasonic: IrriSafe Ultrasonic System 5.5 W, 30 kHz (Satelec Acteon Group, Mérignac, France).

Group C—Sonic: Eddy System 28 mm polyamide tip, 6000 Hz (VDW, Munich, Germany).

Group D—XP-Finisher: 1000 rpm, 1 Ncm (FKG Dentaire, La Chaux-de-Fonds, Switzerland).

Group E—Conventional syringe irrigation (27-gauge needle) as positive control.

Each group was subdivided into two subgroups based on activation durations, 30 s and 60 s, applied to the same 10 teeth for each group.

 **(iv)**  **Irrigant Activation Protocol:**

**Figure 2:**
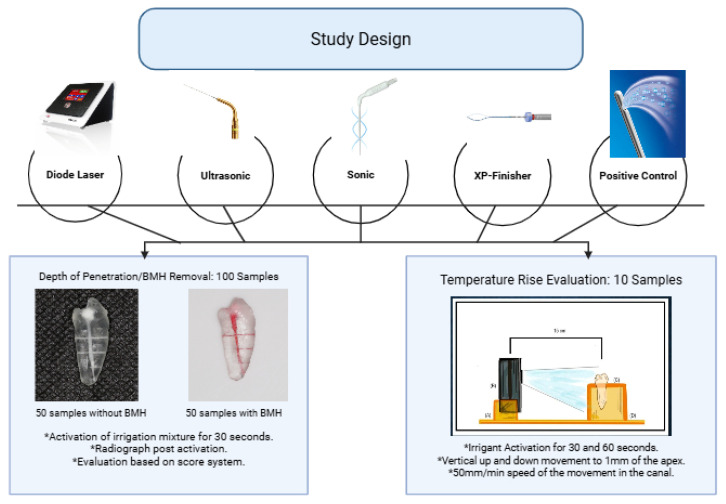
Schematic representation of the study design.* Irrigation activation protocol consistently applied across all samples.

A standardized activation protocol was applied as shown in Figure 2. Canals were filled with 1 mL of 2.5% sodium hypochlorite (NaOCl). A single operator performed all activations to ensure uniformity. The working length was precisely measured and maintained using a rubber stopper to prevent movement beyond the predetermined length, which is 1 mm short of the working length, ensuring consistency across samples. A 7 mm to 8 mm coronal movement at a consistent speed of approximately 50 mm/min, which was approximated through repeated practice using a standardized finger resting technique, guided visually to replicate the controlled, standardized motion typical in clinical endodontic practice. A 10 min cooling period was implemented between tests to mitigate heat retention [29]. Activation duration was timed using a mobile phone stopwatch (iPhone 16 Pro, Apple Inc., Cupertino, CA, USA).

 **(v)**  **Temperature Measurement:**

External root surface temperature rise were recorded using a high-resolution thermal infrared camera; 256 × 192 pixels, 40 mK sensitivity, 0.1 °C resolution, <2% error (TOPDON TC003, Topdon Incorporated, Rockaway, NJ, USA), positioned 15 cm from the sample in designated station to control test environment where external temperature was 23 ± 2. Thermal profiles were captured from 2 s pre-activation to 2 s post-activation. Temperature difference (ΔT) was calculated as the difference between the highest and lowest recorded temperatures. The rate of temperature rise was determined by averaging the temperature increase every 5 s per group for all the samples. Calibration was performed using non-contact infrared thermometers (Fluke 62 MAX, Fluke Corporation, Everett, WA, USA), with emissivity set to 0.96 for consistent readings.

**(II) Biofilm mimicking Hydrogel (BMH) removal of simulated root canal system in 3D-printed premolars:**

 **(i)**  **Sample size calculation:**

The sample size was determined using an online calculator (http://statulator.com/SampleSize/ss2M.html, accessed on 17 May 2025) with an alpha value of 0.05 and a power of 80%. The means (46.2 and 26.2 units) and pooled standard deviation (14.0 units) were obtained from a relevant previous study (28). The calculator determined that a minimum of 10 teeth per group was required for a two-sample comparison (e.g., XP-Finisher vs. Control) (*n* = 10).

 **(ii)**  **Sample preparation:**

One hundred resin-based 3D-printed mandibular single-rooted premolars were created using CBCT data from a prior sample (length: 23 mm; cross-sectional areas: cervical 22.55 mm^2^, mid-root 14.80 mm^2^, apical 11.03 mm^2^), incorporating a 0.25 mm main canal and 0.15 mm lateral canals designed at 3, 7, and 11 mm from the apex for evaluating irrigant penetration and BMH removal [27].

The process involved scanning the tooth with a 3Shape lab scanner (Copenhagen K, Denmark), exporting the data as an STL file, and using Meshmixer software to design the canals. The models were then printed using an ASIGA 3D printer (ASIGA, Sydney, Australia) with clear resin material (Freeprint, Detax, Ettlingen, Germany). Teeth were divided into five groups based on the activation techniques as mentioned in the previous test (20 artificial teeth per group). All the teeth models have been prepared as previously mentioned before for the natural teeth using vortex rotary files up to 40 0.04 taper. Teeth were placed in an artificial socket made of resin and Polyvinyl siloxane (Zhermack, Zhermack SpA, Badia Polesine, Italy) to mimic PDL. A closed system has been created similar to the model of the previous study [10].

 **(iii)**  **Grouping:**

The tooth models have been divided into two subgroups:

a—depth of irrigation penetration without BMH (*n* = 10).

b—depth of irrigation penetration with BMH (*n* = 10).

**Figure 3:**
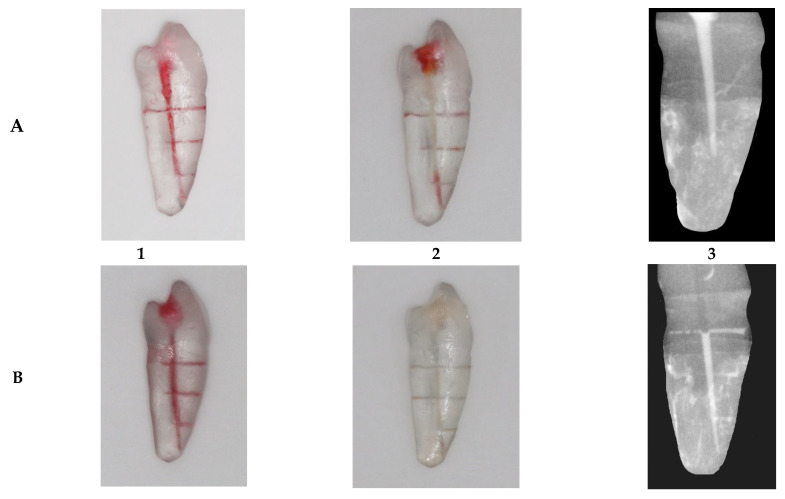
Sample after activation with irrigation mixture ((**A**) 1, 2, 3: laser sample, (**B**) 1, 2, 3: ultrasonic sample), 1: photograph of the sample pre-activation. 2: photograph of the sample post-activation. 3: radiograph of the sample shows distribution of the irrigation mixture within the simulated root canal system.

BMH has been prepared and injected into the canal, based on a previous study [27]. The BMH was created by dissolving 3 g of gelatin (Green, Interlink Foods Ltd., London, UK) and 0.06 g of hyaluronan 95% purity (Thermo Scientific Chemicals, Thermo Fisher Scientific, Swindon, UK) in 45 mL of distilled water at 50 °C. Red food dye was added to aid visualization, and the mixture was injected into the main canal of 50 artificial teeth (*n* = 10) (Figure 2). A Photograph has been taken for the sample with BMH pre- and post-irrigation Activation using SDLR Camera (Canon, EOS 200d, Tokyo, Japan) (Figure 3).

 **(iv)**  **Depth of irrigation measurement:**

A sterile contrast medium solution (Omnipaque 300, GE Healthcare, Cork, Ireland) was mixed with 2.5% NaOCl, based on previous work [28]. This radio-opaque mixture allows visualization of the solution in an artificially created root canal system (Figure 3).

1 mL of the irrigant mixture is injected into the teeth model using a side-vented needle. Then the tip of the device of the activation technique group was inserted into the main canal and activation was performed for 30 s as mentioned in the previous test.

PA radiographs were taken post-activation using a CMOS sensor (NanoPix, Eighteenth Corporation, Changzhou, China) after placing the sample in the designed box to ensure a 90-degree angle exposure with the same distance and alignment across all samples.

**Figure 4:**
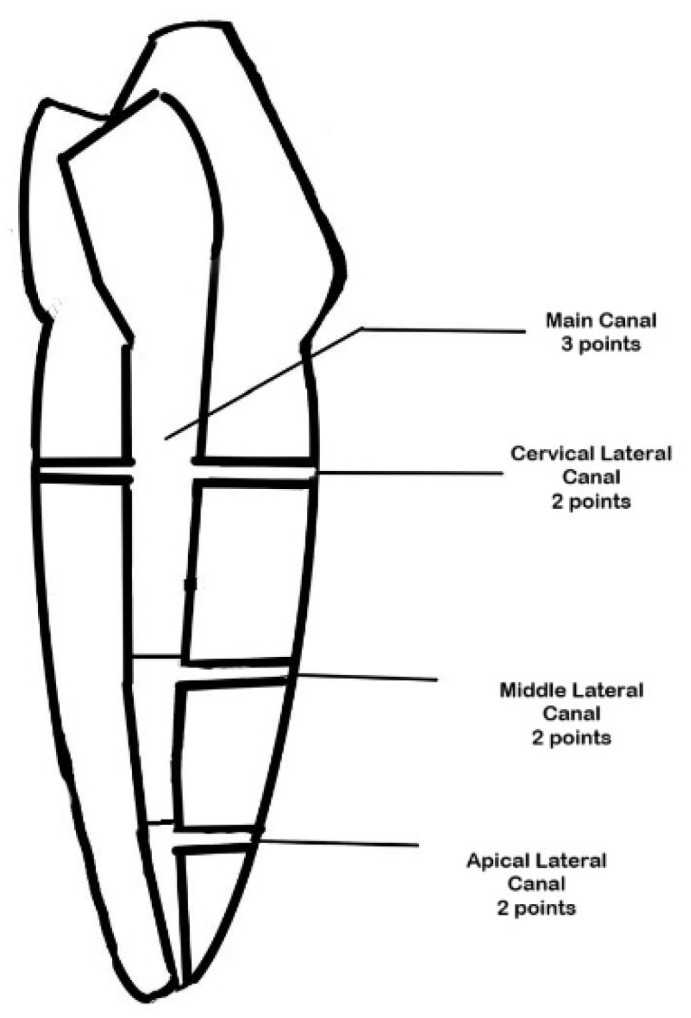
Line diagram illustrating the main canal and lateral canals in the 3D-printed premolars.

The penetration of the contrast medium into the main and lateral canals was assessed using a modified scoring system based on previous studies [5,33] (Figure 4).

Detection of contrast medium in the main canal (coronal, middle, or apical segment) was assigned three points per segment if detected at the level of the lateral canals. (Maximum: three points per main canal if fully penetrated).

Lateral Canals: Detection of contrast medium in each artificial lateral canal was scored as follows: 

Two points: Complete penetration (contrast medium fills more than half of the lateral canal).

One point: Partial penetration (contrast medium present less than half of the lateral canal).

Zero points: No penetration (no contrast medium detected).

Penetration percentage: The score for the sample was converted to a percentage using the following formula:

(Total Points Scored/Maximum Possible Score) × 100 = Penetration Percentage

**Validation of the reliability of the modified scoring system:**

Two endodontists and one endodontic resident independently evaluated periapical (PA) radiographs of 20 main canals and 60 lateral canals. Cohen’s Kappa analysis, using SPSS software, was employed to assess their agreement beyond chance. The Kappa value for the main canal was 0.94, indicating almost perfect agreement, while the lateral canals had a value of 0.85, suggesting substantial agreement. The overall Kappa was 0.89, showing high consistency among evaluators, which reflects the reliability of the modified scoring system.

Following validation of the modified scoring system, which demonstrated high inter-evaluator agreement, a single trained, blinded evaluator scored all radiographic images in the main study. This decision was derived from the substantial to almost perfect agreement to ensure reliability while minimizing scoring variability from multiple evaluators.

**Statistical Analysis:**

All analyses were performed using SPSS (version 24, IBM, Armonk, NY, USA). Due to non-normal data distributions (Shapiro–Wilk, *p* < 0.05), non-parametric tests were applied.

Temperature differences (°C): Friedman tests assessed differences in temperature rise (°C) across five irrigation techniques at 30 s and 60 s. Wilcoxon signed-rank tests (Bonferroni-adjusted α = 0.005) compared time points. The rate of temperature rise was recorded using medians and IQRs, with 95% confidence intervals estimated. Between-group comparisons used Wilcoxon rank-sum tests (Bonferroni-adjusted α = 0.005).

Irrigation Depth of Penetration without BMH/with BMH: Kruskal–Wallis tests compared total scores (0–100%) between groups. Significant results were followed by Bonferroni-corrected Dunn’s tests (α = 0.0125) to compare key pairs. 

Effect sizes for Kruskal–Wallis tests were estimated using η^2^ = (H − k + 1)/(n − k). (H is the test statistic, k is the number of groups, and n is the sample size). Interpretation followed Cohen’s guidelines (η^2^ ≈ 0.01 = small, 0.06 = moderate, 0.14+ = large) [34].

Ordinal regression assessed the influence of activation technique, temperature, and BMH on total scores. Spearman’s correlations (α = 0.01) evaluated relationships between temperature rise and penetration/removal scores. 

## 3. Results

**I: Temperature rise:**

The Friedman test revealed significant differences in temperature rise (°C) across five activation techniques (Diode Laser, Ultrasonic, Sonic, XP-Finisher, Control) at 30 and 60 s (*p* < 0.001, Table 2). Kruskal–Wallis tests showed significant between-group variation at 30 s (H = 44.65, *p* < 0.001, η^2^ = 0.903, 95% CI [0.82, 0.95]) and 60 s (H = 44.25, *p* < 0.001, η^2^ = 0.894, 95% CI [0.81, 0.94]), indicating large effect sizes.

Temperature rise differences (°C) across five activation techniques (Diode Laser, Ultrasonic, Sonic, XP-Finisher, Control) at 30 and 60 s showed significant variation (Table 2). At 30 s, Diode Laser exhibited the highest temperature rise (4.50 °C, IQR: 3.95–5.22), which was significantly higher than the other groups. While the control achieved the lowest temperature rise (0.28 °C, IQR: 0.25–0.32) with no statistically significant differences with Sonic (0.47 °C, IQR: 0.38–0.56), Ultrasonic (0.920 °C, IQR: 71–1.14) or XP-Finisher (0.68 °C, IQR: 0.54–0.83) groups. At 60 s Diode Laser demonstrated the highest temperature rise (8.00 °C, IQR: 7.27–8.86), which was statistically significant with all groups followed by Ultrasonic (2.10 °C, IQR: 1.70–2.58) with significant difference with Sonic (0.52 °C, IQR: 0.43–0.62) and Control (0.33 °C, IQR: 0.31–0.36). 

The rate of temperature rise (°C/s) varied significantly over 60 s. The rate of temperature rise for Diode Laser (0.133 °C/s, 95% CI: 0.121–0.148) was significantly greater than the other groups. While Ultrasonic showed a higher rate of temperature rise significantly than Sonic (0.009 °C/s, 95% CI: 0.008–0.010; *p* < 0.05) and Control (0.036 °C/s, 95% CI: 0.030–0.043; *p* < 0.01), with no significant difference with XP-Finisher (0.021 °C/s, 95% CI: 0.018–0.025; *p* > 0.05).

**Irrigation Penetration without BMH: (** Table 3 **).**

The Kruskal–Wallis test revealed significant differences in irrigation penetration percentages without BMH across groups (H = 11.50, *p* = 0.022, η^2^ = 0.167, 95% CI [0.05, 0.30]), indicating a moderate effect size.

Sonic (88%, IQR: 81–95%) and Ultrasonic (87%, IQR: 81–93%) demonstrated better irrigation penetration, which was statistically significant compared to Diode Laser (79% IQR:72–87%) and Control (65% IQR: 55–78%). No statistically significant difference was found between Sonic, Ultrasonic, and XP-Finisher (68%, IQR: 60–77%).

**Irrigation Penetration with BMH: (** Table 3 **).**

The Kruskal–Wallis test showed significant differences in irrigation penetration percentages with BMH across groups (H = 30.45, *p* < 0.001, η^2^ = 0.588, 95% CI [0.40, 0.75]), indicating a large effect size.

Sonic (83%, IQR: 66.66–88.88%), Ultrasonic (78%, IQR: 66.66–88.88%), and XP-Finisher (63%, IQR: 55.55–66.66%) achieved better BMH removal than Diode Laser (36%, IQR: 27–45%) and Control (33%, IQR: 33.33–44.44%) which was statistically significance. No significant differences were found among Sonic, Ultrasonic, and XP-Finisher or between Control and Diode Laser.

**Ordinal regression of the factors affecting irrigation depth of penetration** showed significant effects for Sonic (β = 2.5, *p* < 0.001), Ultrasonic (β = 2.0, *p* < 0.001), XP-endo Finisher (β = 1.2, *p* = 0.003) vs. Control. Temperature rise (β = −0.1, *p* = 0.505), BMH presence (β = −0.3, *p* = 0.317), and interactions (*p* ≥ 0.230) were non-significant. Spearman’s correlations (α = 0.01) showed no significant temperature rise in the irrigation depth of penetration with BMH or without BMH (without BMH: ρ = −0.15, *p* = 0.32; with BMH: ρ = −0.10, *p* = 0.45).

## 4. Discussion

This study aims to investigate the effect of using five different activation techniques during irrigation activation on temperature rise and irrigation depth of penetration, without BMH/with BMH. The device (Dual wavelength Diode Laser) included in the study presets an endodontic mode; however, no established protocol or data on its thermal safety has been published prior to this investigation. Given the absence of data on temperature rise associated with the simultaneous use of 810 nm + 980 nm wavelengths in continuous lasing within root canals, evaluating the temperature rise in this device was deemed essential.

Temperature rise caused by irrigation activation techniques has been reported in many studies using different temperature measuring methodologies. Thermocouples have traditionally been used to measure temperature changes on the outer root surface. This technique involves two metal connectors exposed to temperature fluctuations. Although thermocouples provide accurate readings, they only measure temperature changes at specific contact points, limiting their ability to detect heat distribution over a broader area [35]. To overcome this limitation, infrared thermography has been explored as an alternative. In 1996, Biagioni et al. introduced digitized infrared thermography to monitor temperature variations on the skin surface overlying the TMJ and masseter muscle during functional activity [36]. Later, in 2000, McCullagh et al. [37] investigated temperature rises on the outer surface of single-rooted teeth during continuous wave obturation using both infrared thermography and thermocouples. Their study demonstrated that infrared thermography can effectively map temperature rise across broader areas beyond the limited scope of thermocouples.

The experimental model in this study was adapted from prior research evaluating the irrigation activation depth of penetration within the root canal system [10,27,28,33]. In some studies, large endodontic instruments were used to create lateral canals for assessing dye penetration depth, which can introduce variability and impact test reliability. In this study, lateral canals were 3D-printed with a diameter of 0.15 mm (150 µm), similar to that of the natural lateral canal shape and size [38,39], ensuring high standardization. Additionally, the use of a contrast medium with an imaging technique, as employed in previous studies [10,28], enhanced visualization of irrigant distribution and penetration depth within the simulated root canal system without removing the sample from the artificial socket, which can affect the fluid dynamics of the irrigant within the sample and negatively influence the results. 

This study found that continuous lasing with a dual-wavelength (810 nm + 980 nm) diode laser at 0.7 W caused a linear temperature rise on the outer root surface, approaching the 10 °C safety limit after 60 s of irrigant activation, consistent with previous studies [40,41,42]. Moreover, with temperature rises limited to 10 °C, it is considered well below thresholds for dentin degradation (>200 °C) and within dentin thermal tolerance [43]. 

The power output and mode of lasing are critical factors influencing thermal safety during irrigation activation and canal disinfection. Temperature rise on the external root surface increases proportionally with increased laser power settings [40]. While continuous mode showed to be associated with thermal elevation as in our study, the literature suggests that using pulsed mode, especially at lower power settings, can significantly reduce thermal effects [44,45]. 

Diode Laser in this study achieved moderate irrigation penetration depth without BMH compared to control, and this can be attributed to vertical movement within the canal, which facilitated better fluid hydrodynamic and BMH removal (10). With in-depth penetration with BMH, a Diode Laser showed lower results than Sonic, Ultrasonic, and XP-finisher, and this may be due to a lack of cavitation within the irrigation, which has been reported previously [46].

In this study, Ultrasonics showed lower temperature rise than a Diode Laser at 30 and 60 s. The duration of the Ultrasonic activation technique resulted in a linear temperature rise, which has been addressed in the literature [16]. This may be due to the friction of the tip against the canal wall and from direct conversion of sound energy into heat, which creates cavitation [47]. 

Cavitation and acoustic streaming of Ultrasonic and Sonic irrigation activation techniques can be hypothesized for their superior overall performance in the irrigation depth of penetration with BMH and without BMH. This aligns with previous studies reporting comparable performance of Sonic and Ultrasonic [48,49]. 

In this study, it was observed that XP-Finisher’s irrigation depth of penetration was unaffected with or without BMH as Sonic and Ultrasonic irrigation activation techniques. Its ability to remove debris and root canal filling remnants, particularly in the apical third, has been attributed to its transformation and expansion within the root canal system [50].

Irrigation activation was performed at room temperature (23 + 2 °C) for 30 s to assess irrigant penetration depth, assisted by the selected techniques under standardized conditions. Although a slight temperature rise was observed during activation, it did not appear to affect the flow of the irrigant within the simulated root canal system. This is consistent with previous research, which reported that increasing the temperature of NaOCl from 22 °C to 37 °C did not significantly influence its flow characteristics [51]. Furthermore, it has been shown that prolonged contact time and higher NaOCl concentrations play a more critical role in enhancing its penetration into dentinal tubules, primarily by improving its flow properties [52].

**Clinical Significance:**

This study highlights the clinical significance of temperature rise and irrigation activation efficacy in an in vitro setting. Diode Laser (810 + 980 nm) caused a significant temperature rise within the experimental durations, but it did not exceed the safety limit of 10 °C as all irrigation activation techniques tested in the current study. Sonic and Ultrasonic techniques demonstrated superior irrigation depth of penetration without BMH/with BMH supporting their superiority in creating better irrigation distribution within the root canal system.

**Limitations:**

The findings of our study on Diode Laser-assisted irrigation activation must be interpreted with caution regarding both efficacy and safety, as they are limited by the specific parameters employed, particularly the use of a continuous lasing mode at a fixed wavelength and power, which restricts their generalizability to other settings or laser systems, underscoring the need for careful interpretation. Moreover, Diode Lasers have proven effective in various treatment applications [53,54]. New advancements in Laser treatment, including Ultra-Fast Femtosecond Laser irradiation, have emerged as effective in ablating enamel and dentin with high precision and minimal collateral thermal stress, which can be investigated in terms of irrigant activation to evaluate its effectiveness [55].

This in vitro study, designed to evaluate fluid dynamics in a simulated clinical scenario using a closed system with main and lateral canals, has limitations that affect its generalizability. The use of 3D-printed clear resin models and extracted human teeth, selected for reproducibility, differs from vital teeth in material properties, potentially affecting irrigant flow and (BMH) removal. While providing a controlled environment, these models do not account for physiological factors such as blood perfusion or dentinal fluid, which may influence thermal dissipation and irrigant behavior.

The in vitro setup, including BMH, does not replicate the natural biofilm complexity or patient-specific factors like canal anatomy variations, limiting clinical applicability.

Additionally, cross-over thermal bias may occur due to residual heat accumulation despite a 10 min cooling period, potentially affecting temperature rise data for techniques like the Diode Laser. 

This study did not assess the bacterial reduction capabilities of the irrigation activation techniques. Future studies should focus on evaluating microbial reduction to better assess the efficacy of these systems, incorporating simulated blood flow to more accurately mimic clinical conditions. Additionally, investigating various Diode Laser settings (e.g., power and pulse duration) is recommended to enhance clinical relevance.

## 5. Conclusions

Within the limitations of this in vitro study, all irrigation activation techniques resulted in a temperature rise within a clinically acceptable range. However, Diode Laser, Ultrasonic, and XP-Finisher caused linear temperature rise with activation time. Sonic and Ultrasonic techniques demonstrated superior irrigant penetration depth, with and without BMH, compared to other activation techniques.

## Figures and Tables

**Table 1:** Average of dentinal thickness of the selected samples in mm.

Root Level	Root (B-L)	Root (M-D)	Lumen (B-L)	Lumen (M-D)
Cervical	6.65 ± 0.263	4.32 ± 0.285	2.98 ± 0.226	1.50 ± 0.210
Middle	5.83 ± 0.221	3.24 ± 0.227	2.10 ± 0.258	1.26 ± 0.134
Apical	5.03 ± 0.383	2.80 ± 0.124	1.40 ± 0.182	0.90 ± 0.133

**Table 2:** Temperature rise on outer root surface in Celsius.

Method	30 Seconds (°C) Median (IQR)	60 Seconds (°C) Median (IQR)	*p*-Value (30 s vs. 60 s)
Control	0.28 (0.25–0.32) ^a^	0.33 (0.31–0.36) ^ae^	0.096
Diode Laser	4.50 (3.95–5.22) ^abcd^	8.00 (7.27–8.86) ^abcdf^	0.001 *
Ultrasonic	0.92 (0.71–1.14) ^b^	2.10 (1.70–2.58) ^bef^	0.001 *
Sonic	0.47 (0.38–0.56) ^d^	0.52 (0.43–0.62) ^c^	0.547
XP-Finisher	0.68 (0.54–0.83) ^c^	1.22 (0.96–1.48) ^d^	0.007 *
*p*-value (between groups)	<0.001 *	<0.001 *	

* Significance was considered at *p* < 0.05. Superscript letters (a, b, c, etc.) indicate significant differences between groups in the same column.

**Table 3:** Comparison between the groups in terms of irrigation penetration with BMH and without BMH in percentage.

Method	Without BMH (%) Median (IQR)	With BMH (%) Median (IQR)	*p*-Value (Without vs. With BMH)
Control	65 (55–78) ^ac^	33 (33.33–44.44) ^abc^	0.0045 *
Diode Laser	79 (72–87) ^bd^	36 (27–45) ^def^	0.0005 *
Ultrasonic	87 (81–93) ^ab^	78 (66.66–88.88) ^ad^	0.121
Sonic	88 (81–95) ^cd^	83 (66.66–88.88) ^be^	0.122
XP-Finisher	68 (60–77)^f^	63 (55.55–66.66) ^cf^	0.51
*p*-value (between groups)	0.022 *	<0.001 *	

* Significant at *p* < 0.05 (between groups) or *p* < 0.01 (within groups). Superscript letters (a, b, c, etc.) indicate significant differences between groups in the same column.

## Data Availability

The dataset analyzed during this article is available from the first author upon request.

## Abbreviations

The following abbreviations are used in this manuscript:

BMH	Biofilm-Mimicking Hydrogel
PDL	Periodontal ligament
